# Nano-thick calcium oxide armed titanium: boosts bone cells against methicillin-resistant Staphylococcus aureus

**DOI:** 10.1038/srep21761

**Published:** 2016-02-22

**Authors:** Huiliang Cao, Hui Qin, Yaochao Zhao, Guodong Jin, Tao Lu, Fanhao Meng, Xianlong Zhang, Xuanyong Liu

**Affiliations:** 1 State Key Laboratory of High Performance Ceramics and Superfine Microstructure, Shanghai Institute of Ceramics, Chinese Academy of Sciences, Shanghai 200050, China; 2 Department of Orthopedics, Shanghai Sixth People’s Hospital, Shanghai Jiao Tong University, Shanghai 200233, China

## Abstract

Since the use of systemic antibiotics for preventing acute biomaterial-associated infections (BAIs) may build up bacterial resistance and result in huge medical costs and unpredictable mortality, new precaution strategies are required. Here, it demonstrated that titanium armed with a nano-thick calcium oxide layer was effective on averting methicillin-resistant Staphylococcus aureus (MRSA) infections in rabbits. The calcium oxide layer was constructed by, firstly, injecting of metallic calcium into titanium via a plasma immersion ion implantation process, and then transforming the outer most surface into oxide by exposing to the atmosphere. Although the calcium oxide armed titanium had a relative low reduction rate (~74%) in growth of MRSA *in vitro*, it could markedly promote the osteogenic differentiation of bone marrow stem cells (BMSCs), restore local bone integration against the challenge of MRSA, and decrease the incidence of MRSA infection with a rate of 100% (compared to the titanium control). This study demonstrated for the first time that calcium, as one of the major elements in a human body, could be engineered to avert MRSA infections, which is promising as a safe precaution of disinfection for implantable biomedical devices.

Biomaterial-associated infections (BAIs) were lethal and costly complications to those increasingly used implantable biomedical devices^1^,^2^,^3^, some of which were made from titanium based materials^4^. It was reported that over 5% of the dental implants may become infected^5^, and this incidence for fixing bone fractures with open wounds may exceed 30%^6^. The high rate of BAIs gives ~55,000 deaths annually in the United States alone, with similar morbidities worldwide^7^. The average revision costs were many times more than that of a primary insertion^8^.

Generally, BAIs were resulted by adhesion of pathogenic microbes and subsequent formation of biofilms^9^. Antibiotics were taken as standard precautions against bacterial contaminations, but they faced severe challenges in the crisis of drug resistance, which saw the worldwide spread of persisters, such as methicillin-resistant Staphylococcus aureus (MRSA). It was reported that over 50% of the Staphylococcus aureus (S. Aureus) mediated orthopedic infections, admitted to the Shanghai Sixth People’s Hospital (in China) during 2006 to 2011, were resistant to methicillin^10^. Moreover, it was realized that colonization of bacteria was not the only process responsible for the high susceptibility of biomaterials to infections. Simply 100 colony-forming units (CFU) of Staphylococcus aureus (S. aureus) were sufficient to infect 95% of the subcutaneous implants, though 10^5^-fold higher of that did not produce any abscesses in the absence of alien materials^11^. The surgical site inserted of an arthroplastic implant could become infected even by contaminating of less than 50 CFU of S. aureus, which was 200 times lower than that in the absence of the foreign device^12^. These results were mainly due to the undesirable host responses, which compromises the regional capability in bacterial clearance^13^,^14^. Therefore, engineering biomaterial surfaces with improved biocompatibility, which can restore the functions of cells around the indwelt device and accelerate local tissue integration, may be a promising direction in combating BAIs^15^,^16^.

Calcium (Ca) has the most pronounced effects on modulating of bone mass^17^, even at high concentrations (~10 mM)^18^, whereas calcium homeostasis should be tightly controlled by bacteria (ranging from 100 to 300 nM) in case of undue accumulation^19^,^20^. These facts indicated that calcium may be used to engineer biomedical devices of selective toxicity, i.e. inhibites bacterial adhesion but promotes osteogenesis. It was evidenced that Ca-modified titanium has good biocompatibility to bone cells *in vitro*^21^,^22^,^23^ that encouraged osseointegration *in vivo*^24^,^25^, whereas, the modification also increased the adhesion of bacterial cells^26^, which compromises the clinical use. Nevertheless, it was revealed that calcium in oxide or hydroxide forms was efficient antibacterial agents^27^,^28^,^29^, indicating that control the form of calcium in titanium may improve its performance against bacteria. Accordingly, in this study, a nano-thick calcium oxide layer was constructed on titanium surfaces, and we found that it could avert MRSA(ATCC 43300) infection by restoring the osteogenic functions of bone marrow stem cells (BMSCs) and promoting local bone tissue integration in rabbits.

## Results

### Fabrication of the calcium oxide layer

**Figure 1:**
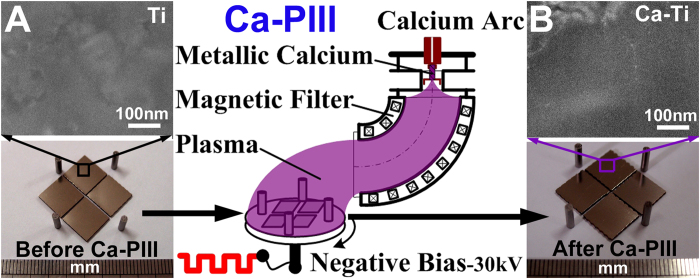
Surface morphology of the titanium before (Ti, **A**) and after undergoing calcium acr sourced plasma immersion ion implantation (Ca-Ti, **B**).

**Figure 2:**
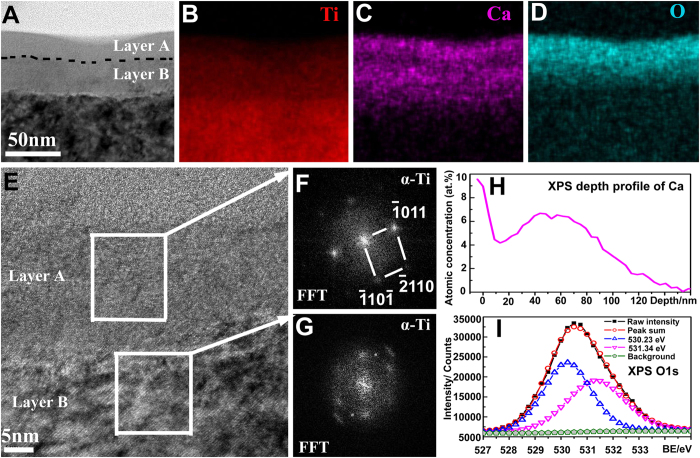
Structure of the calcium oxide layer: cross-sectional transmission electron microscopy (TEM), bright field image (**A**), scanning transmission electron microscopy-energy dispersive X-ray spectroscopy (STEM/EDS) mapping of Titanium (**B**), Calcium (**C**), Oxygen (**D**), and high resolution TEM image (**E**) with the corresponding fast Fourier transform (FFT) patterns (**F,G**); X-ray photoelectron spectroscopy (XPS) depth profile of Calcium (**H**) and the O1s peak (**I**).

The calcium oxide layer was fabricated by a two-step procedure. Firstly, the polished titanium (designated as Ti, Fig. 1A) was treated by a calcium arc sourced plasma immersion ion implantation (Ca-PIII) at 30 kV for 90 min to introduce metallic calcium into the substrate surface. During Ca-PIII, a pure calcium cathode was triggered to produce a plasma, which contained positively charged calcium ions that were accelerated by and injected into the negatively biased titanium samples. Secondly, the Ca-PIII treated samples were further exposed to atmosphere (for over 72 h) to oxidize the outer most surfaces of the layer stored of metallic calcium (designated as Ca-Ti, Fig. 1B). Cross-sectional transmission electron microscopy (TEM) analysis demonstrated that a calcium reservoir of about 68 nm in thickness was conjugated to the titanium substrate (Fig. 2A). The reservoir comprised two distinguished sub-layers, layers A and B. Scanning transmission electron microscopy-energy dispersive X-ray spectroscopy (STEM/EDS) mapping (Fig. 2B–D) revealed that both the sub-layers contained calcium, but the outer layer (layer A) was apparently oxidized due to atmosphere exposure. Although titanium crystals in both layers were separated into small segments and became partially amorphous (Fig. 2E), typical fast Fourier transform (FFT) patterns of close-packed hexagonal α-Ti still was resolved (Fig. 2F,G). The highest calcium concentration determined by X-ray photoelectron spectroscopy (XPS) reached 9.6 at.% at the outer surface (Fig. 2H), and the XPS O1s peaks at 530.23 eV and 531.34 eV corresponded to titanium oxide and calcium oxide (Fig. 2H)^30^, respectively. The result was in consistence with STEM/EDS mapping results (Fig. 2B–D). Furthermore, the concentration of calcium liberated by the Ca-Ti group, determined by inductively-coupled plasma optical emission spectrometry (ICP-OES), was about 750 nM (per square centimeter) in the first day, and it maintains such levels for at least 28 days (the longest period evaluated).

### *In vitro* responses of bacterial and mammalian cells

**Figure 3:**
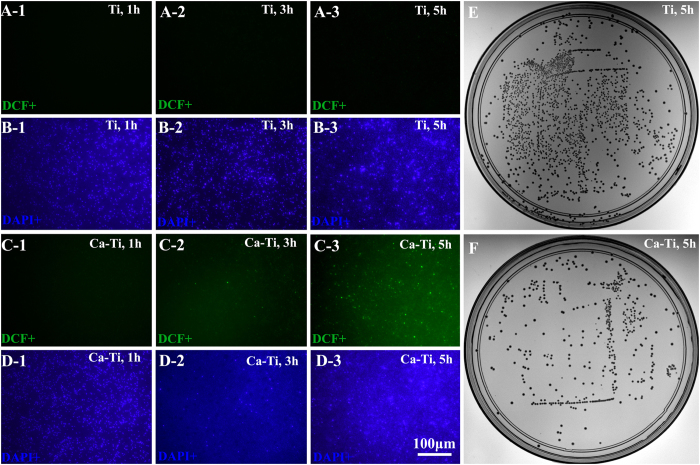
*In vitro* anti-MRSA activity of the calcium oxide armed titanium: the bacterial cells, after culturing on the samples for 1(i-1), 3(i-2), and 5 h(i-3), were counter-stained with DCFH-DA(transfered to DCF of green fluorescence, indicating the ROS levels in microbes ) and DAPI(blue, indicating the total amount of adherent bacteria). For i = A and B correspond to Ti group; i = B and C correspond to Ca-Ti group. The adherent bacteria, after cultured for 5 h, were dissociated and recultured on tryptone soya broth (TSB) agar plates to show the amount of viable microbes(E for Ti, F for Ca-Ti).

**Figure 4:**
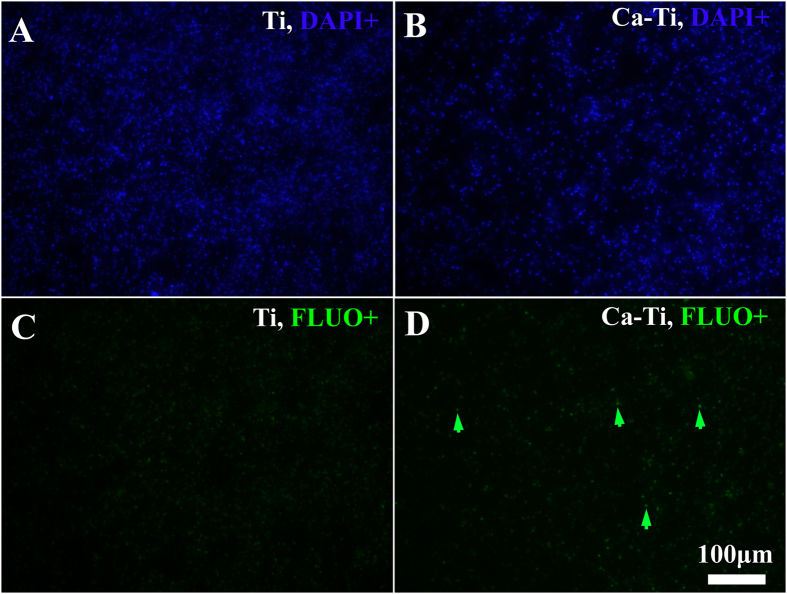
Calcium concentration levels imaged by Fluo-3 AM/DAPI counter staining assay: (**A**,**B**) were the DAPI positive(DAPI+) bacteria, (**C**,**D**) were the corresponding FLUO positive(FLUO+). FLUO(green) indicated the intracellular calcium levels, while DAPI(blue) indicated the bacterial chromosome(the total amount of the adherent bacteria).

*In vitro* anti-MRSA activity was evaluated by inoculating methicillin-resistant *Staphylococcus aureus* (MRSA, ATCC43300) onto the ultraviolet sterilized samples and incubating at 37 °C for various durations. The persistent nature of the bacterial strain was confirmed by the results obtained via the standardized disc susceptibility testing method (Fig. S1, Supporting Information)^31^. As elevating the intracellular reactive oxygen species (ROS) levels was a common pathway quickly poison bacteria^32^. And oxidation of non-fluorescent 2**′**,7**′**-dichlorofluorescin-diacetate (DCFH-DA) to luminous 2**′**,7**′**-dichlorofluorescein (DCF, green fluorescence) provided an effective procedure for detecting ROS formation^33^. The bacterial cells, after culturing on these samples for 1, 3, and 5 h, were counter-stained with DCFH-DA and 4**′**-6-diamidino-2-phenylindole (DAPI, blue fluorescence) to trace the ROS levels and calculate the ratios of DCF positive (DCF^+^) cells. As shown in Fig. 3, the intensity of the green fluorescence on Ca-Ti (Figs 3C-2 and 3) was apparently stronger than that on the pure Ti (the control, Figs 3A-2 and 3), revealing that the ROS levels for the former were higher than that in the latter. Furthermore, it was found that, after a 5 h-cultrue, 62.8% of the bacteria on Ca-Ti became DCF^+^(Fig. 3C-3, the corresponding DAPI positive, DAPI^+^ images were shown in Fig. 3D-3), while that for Ti was only 0.7%, indicating Ca-Ti was good at stimulating generation of ROS. The adherent bacteria, after cultured for 5 h, were dissociated and re-cultured on tryptone soya broth (TSB) agar plates to show the amount of intact cells. The results indicated that Ca-Ti could apparently inhibit the growth of MRSA in a reduction rate of 74% (Fig. 3F, the Ti control was in Fig. 3E). Since, over accumulation of calcium was lethal to microbes^20^, the accumulation of calcium in the adherent bacteria was imaged according to the Fluo-3 AM (green fluorescence)/DAPI(blue fluorescence) counter staining assay. As shown in Fig. 4, only small amount of bacterial cells, compared with the Ti control, were in higher levels of calcium concentration (arrowed in Fig. 4D). And the percentage of the Fluo positive (Fluo^+^, green) cells for Ca-Ti was about 32.9%, which was merely half of the rate ROS (62.8%), indicating that calcium accumulation was not the sole action of Ca-Ti to bacteria.

**Figure 5:**
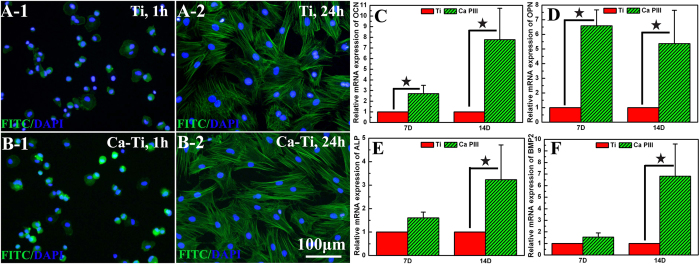
Biocompatibility of the calcium oxide armed titanium: after culturing the bone marrow stem cells (BMSCs) for 1h or 24 h on Ti (**A**-1 or 2) and Ca-Ti (**B**-1 or 2), the cells were counter-stained with FITC-Phalloidin (green, showing the F-actin) and DAPI (blue, indicating the nuclei); after culturing the BMSCs for 7 or 14 days, the gene expression of osteocalcin (OCN, **C**), osteopontin (OPN, **D**), alkaline phosphates (ALP, **E**), and bone morphogenetic protein 2 (BMP2, **F**) were determined by real-time polymerase chain reaction (RT-PCR, *p < 0.05).

Moreover, the *in vitro* biocompatibility of Ca-Ti was evaluated by using the bone marrow stem cells (BMSCs). The results shown that the embedded calcium oxide layer did no harm to the initial adhesion and spreading of BMSCs (Figs 2 and 5B-1). In addition, up-regulated activities in expression of osteocalcin (OCN, Fig. 5C), osteopontin (OPN, Fig. 5D), alkaline phosphates (ALP, Fig. 5E), and bone morphogenetic protein 2 (BMP2, Fig. 5F) were observed by incubating the cells onto Ca-Ti and culturing for 7 and 14 days, respectively, indicating that Ca-Ti enhanced the osteoblastic differentiation of BMSCs.

### *In vivo* anti-MRSA property

**Figure 6:**
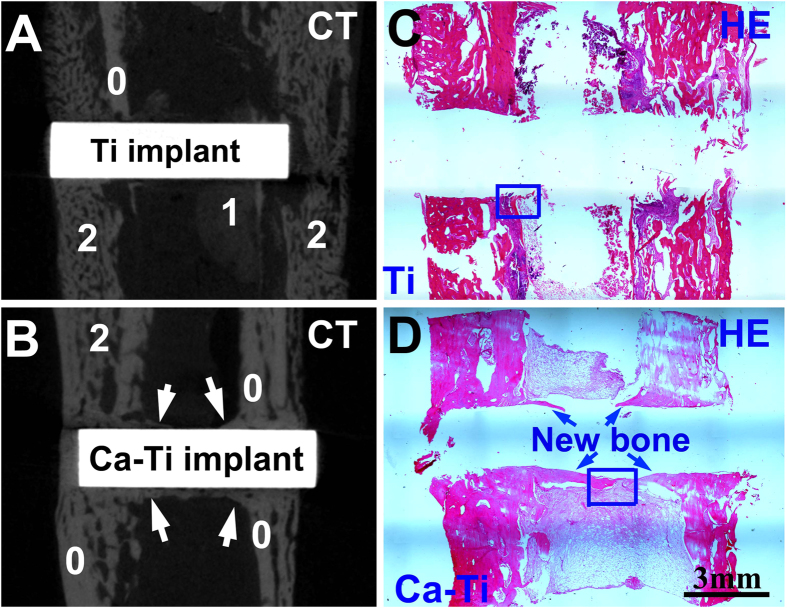
*In vivo* effect of the calcium oxide armed titanium on MRSA infection: micro-computed tomography (Micro-CT) images acquired around Ti (**A**) and Ca-Ti (**B**) implants, which were contaminated by MRSA and settled in a rabbit tibia for six weeks. The numbers, 0 for the original tibia, 1 for sequestrum, 2 for periosteal new bone, respectivley; Hematoxylin & eosin and Giemsa staining of the corresponding sagittal sections for Ti (**C**) and Ca-Ti (**D**, the new bone was arrowed) at low magnifications.

**Figure 7:**
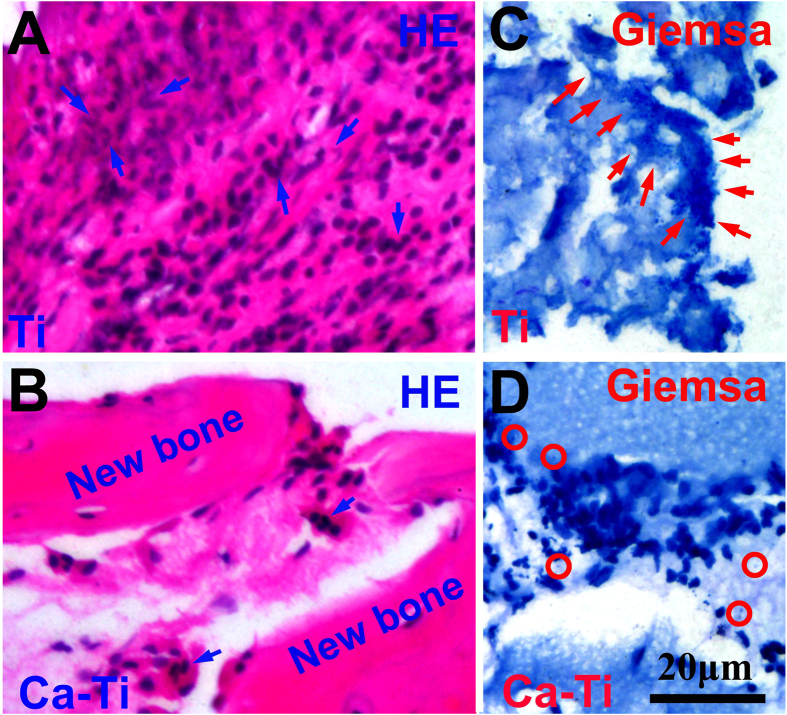
*In vivo* effect of the calcium oxide armed titanium on MRSA infection: Hematoxylin & eosin and Giemsa staining of the corresponding sagittal sections for Ti (**A**, the segmented neutrophils were arrowed) and Ca-Ti (**B**); Giemsa staining of the corresponding sagittal sections for Ti (**C**, as arrowed in the figure, the bacteria gather together), Ca-Ti (**D**, as circled in the figure, the bacteria were isolated from one another). These were the high magnification images corresponding to the circled areas in Fig. 6C,D, respectively.

The effect of the calcium oxide layer on preventing BAI was evaluated via an infection model in rabbits (Fig. S2, supporting information), in which a Ca-Ti together with a Ti control were implanted in the same tibia to exclude the host difference between the animals (n=6, one of them was not contaminated with bacteria; the other five were injected of MRSA). The symptoms of BAI during the six weeks in housing the animals were recognized by X-ray radiograph. Without bacterial contamination, both the implants had no sign of infection during the whole housing. However, with the challenge of MRSA, serious periosteal reactions around the Ti implants were evidenced at the second week after the surgery, whereas there was no obvious evidence for Ca-Ti until the sixth week. This was consistent with appearance of the whole corresponding tibia (obtained at the sixth week), showing that periosteal new bone formation in the sites where the Ti samples implanted were evident. The gross tibia was further scanned by a micro-computed tomography system (Micro-CT). As the results shown in Fig. 6, sequestrum and periosteal new bone were formed because of serious infections next to Ti (Fig. 6A), but good osseointegration was constructed on Ca-Ti (arrowed in Fig. 6B). The evolutions in histological morphology between the sample groups were assessed by hematoxylin & eosin staining assay. As shown by the panoramic images, formation of healthy new bone on the surface of Ca-Ti implants was detected (Fig. 6D), while periosteal reaction was serious next to the Ti implant (Fig. 6C). In addition, large flows of inflammatory cells, such as band and segmented neutrophils (arrows in Fig. 7A), were recruited to the Ti surfaces, whereas they were seldom detected near the Ca-Ti (Fig. 7B), indicating that the battle to MRSA was terrible at Ti but a landslide victory of Ca-Ti. This evidence was consistent with the result obtained by Giemsa staining. It was found that the bacterial cells around Ca-Ti were isolated from one another (Fig. 7D), while that around Ti group gathered (Fig. 7C), indicating that the bacteria around Ti implant were more aggressive than that around Ca-Ti implants. Statistics showed that the Ca-Ti group, compared to the titanium control, decreased the incidence of MRSA infection with a rate of 100%, demonstrating good reproducibility.

## Discussion

In the current study, nano-thick calcium oxide was constructed on the titanium surface by using the plasma immersion ion implantation (PIII) process with a mono plasma of calcium (Ca-PIII). After Ca-PIII, the calcium reservoir was exposed to atmosphere to form a typical double-layered structure: the oxidized outer layer and the retained interior layer. The calcium oxide layer embedded in titanium was found effective on averting methicillin-resistant Staphylococcus aureus infection in rabbits.

**Figure 8:**
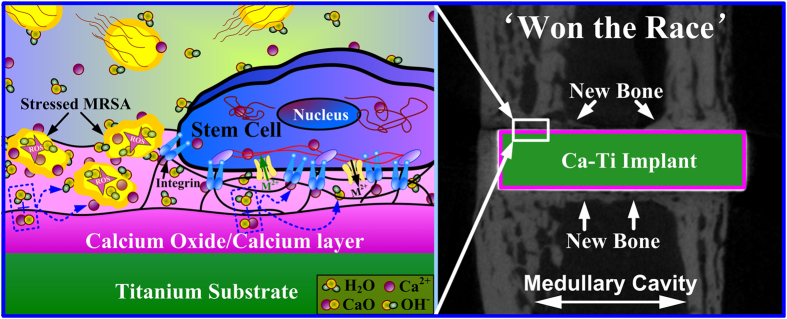
Illustration of the biological actions of the calcium oxide armed titanium: the material releases calcium and hydroxyl ions that not only enhance the generation of reactive oxygen species (ROS) in methicillin-resistant Staphylococcus aureus (MRSA), but also restore the osteogenic functions of bone marrow stem cells (BMSCs) and accelerate local bone integration to combat bacterial colonization.

As presented previously, the reservoir of calcium in titanium did harm to MRSA by promptly enhancing the levels of intracellular reactive oxygen species (ROS, Fig. 2). This was likely due to spontaneous hydration process of calcium oxide in water, during which calcium hydroxide was produced, and hydroxyl ions (OH^−^) were released (local pH increases)^34^,^35^. It was well known that hydroxyl ions were lethal to bacterial cells because of their oxidant activity^29^,^36^. Moreover, calcium ions (Ca^2+^), the other product in hydration of calcium oxide, may make the living environment of bacteria worse. It was revealed that the intracellular concentration of calcium should be tightly regulated by bacteria in a range from 100 to 300 nM^19^ and the calcium liberated by Ca-Ti (~750 nM) was over two times higher than these values. MRSA cells, as encountered the nano-thick reservoir of calcium, were likely to efflux calcium through consuming of adenosine triphosphate (ATP)^37^, and ROS could be a by-product^38^. However, only small amount of bacterial cells were detected in higher levels of calcium concentration (Fig. 4D), indicating that the antibacterial activity of Ca-Ti was dependent on the synergistic effect of calcium and hydroxyl ions (Fig. 8).

Nevertheless, the process was favorable to mammalian cells. As demonstrated throughout this study, the calcium-modified titanium enhances the osteoblastic differentiation of BMSCs (Fig. 5). In fact, bone regeneration was pH dependent, with an optimum level at about pH 8–8.5^39^. On the other hand, calcium had the most pronounced effects on modulating of bone mass^17^, facilitating bone formation and osseointegration even at high concentrations (~10 mM)^18^. In addition, it was well known that neutrophils, the key players in the innate immune system, release calprotectin (a heterodimer of two calcium-binding proteins) to inhibit bacterial superoxide defense and kill of bacteria^40^. And the molecule could be purified in presence of calcium ions (Ca^2+^), guaranteeing the antibacterial activity of neutrophils against S.aureus^41^. As a result, the host actively uses calcium to orchestrate the inflammatory response to a wound^42^. These facts were consistent with our findings *in vivo*, which demonstrated that good osseointegration was constructed on Ca-Ti implants, but serious infections were spoted around Ti implants (Fig. 6). The results clearly proved that the calcium oxide layer embedded in titanium gave bone tissue a clear advantage in winning the race against MRSA.

In summary, a nano-thick calcium oxide layer was conjugated to the titanium surface by, firstly, injecting of metallic calcium into titanium via a plasma immersion ion implantation process, and then transforming the outer most layer into oxide by exposing to the atmosphere. It demonstrated that the calcium oxide layer protected titanium from methicillin-resistant Staphylococcus aureus (MRSA, ATCC 43300) infections not only by enhancing the generation of reactive oxygen species (ROS) in bacteria, but also via restoring the osteogenic functions of bone marrow stem cells (BMSCs) and accelerating local bone integration to combat the invasion by microbes. Since bacterial contamination and undesirable host responses were the two major aspects answer for the susceptibility of an implant to biomaterial-associated infections (BAIs), this dual property of calcium oxide armed titanium is promising for precaution of BAIs. This study gave new insight into designing and fabricating antibacterial medical devices.

## Methods

### Fabrication and characterization of the calcium oxide layer

Polished commercial pure titanium samples (10 mm or 20 mm square plates with a thickness of 1 mm were used for *in vitro* evaluation; cylindrical rods, 7 mm in length and 2 mm in diameter, were used for *in vivo* studying.) were treated by plasma immersion ion implantation, which was sourced by filtered cathodic arc of pure calcium (Ca-PIII). The parameters used in Ca-PIII were detailed in Table 1. After Ca-PIII, the samples were exposed to atmosphere to form a layer of calcium oxide. The cross-sections of the PIII treated samples were investigated by transmission electron microscopy (TEM, FEI Tecnai G2 F20) equiped with STEM/HAADF and EDX Mapping/Line-scan/Probe Spectrum units. The TEM samples were prepared by focused ion beam (FIB, FEI Helios). The chemical states for those concerned elements were determined by X-ray photoelectron spectroscopy (XPS, PHI5300), during which a 14 keV electron gun(250 W) was aimed at an aluminum target to produce the low energy X-ray. To monitor ion release of the Ca-PIII modified titanium group (designated as Ca-Ti), the samples(one 20 mm square plate was used for every group) were incubated in 5 ml physiological saline (0.9% NaCl) at 37 °C for various periods of time without stirring, and the amount of calcium released from the samples into the liquids were determined by inductively-coupled plasma optical emission spectrometry (ICP-OES, Vista AX, USA).

### *In vitro* antibacterial assay

The samples were sterilized by illuminating with ultraviolet light (40 watt) for over 24 h. The methicillin-resistant *Staphylococcus aureus*(MRSA, ATCC43300) suspension (cultured in tryptone soya broth, TSB, OXID LTD,England) with OD_600_ of 0.3 (in the logarithmic phase) was diluted by adding 0.5 ml of the suspension into 4.5 ml physiological saline (0.9% NaCl). Then, 60 μl of the diluted suspension was introduced onto each of the samples(10 mm square plates). The samples with the bacterial suspension were incubated at 37 °C for required durations. For quantitative analysis of the reduction rate, after a 5 h-incubation, the samples were gently rinsed once with physiological saline, and put into each test tube with 5 ml physiological saline. The test tube was vigorously vortexed for over 60 s using a vortex mixer to detach the bacteria from the samples. Subsequently, the suspensions were serially diluted in ten-fold steps with sterile physiological saline. Then 100 μl of the diluted bacterial suspension was inoculated onto TSB agar plates. After incubation at 37 °C for 24 h, the bacterial colonies on Ti and Ca-Ti group were counted (designed as **B**_Ti_ and **B**_Ca-Ti_, respectively), and the reduction rate of Ca-Ti group (**R**_Ca-Ti_) can be calculated according to **R**_Ca-Ti_ = (**B**_Ti_ − **B**_Ca-Ti_)/**B**_Ti_.

### Detection of reactive oxygen species (ROS)

2**′**,7**′**-dichlorofluorescin-diacetate (DCFH-DA) assay was applied to determine the burst of intracellular reactive oxygen species (ROS). The oxidation of non-fluorescent DCFH-DA to fluorescent 2**′**,7**′**-dichlorofluorescein (DCF) provides a procedure for detection of ROS formation^33^. After culturing the bacteria on the samples(10 mm square plates) for 1, 3, and 5 h, the samples together with the bacteria were rinsed once with physiological saline, and stained with 10 mM DCFH-DA (at room temperature for 30 min). Afterwards, the samples were rinsed twice with saline, fixed with 4% paraformaldehyde(PFA, at room temperature for 10 min), and counter-stained with 4**′**-6-diamidino-2-phenylindole (DAPI, 1 μg /ml, at room temperature for 5 min). The percentage of the ROS positive cells was the ratio of DCF^+^/DAPI^+^.

### Imaging the intracellular calcium

The Fluo-3 AM/ DAPI counter staining assay was used to image the calcium ion uptake of the adherent bacterial cells. Stock solutions (1 mM) of Fluo-3 AM (Sigma-Aldrich) were prepared by dissolving in absolute dimethylsulphoxide(Sigma-Aldrich). The aliquots were diluted with physiological saline to give the final concentrations of 10 μM. After culturing the bacteria on the samples(10 mm square plates) for 5 h, the samples together with the bacteria were rinsed once with physiological saline, and stained with 10 μM Fluo-3 AM (at 37 °C for 30 min). Afterwards, the samples were rinsed twice with saline, fixed with 4% paraformaldehyde (PFA, at room temperature for 10 min), and counter-stained with 4**′**-6-diamidino-2-phenylindole (DAPI, 1 μg /ml, at room temperature for 5 min). The percentage of the Fluo positive(Fluo^+^) cells was the ratio of Fluo^+^/ DAPI^+^.

### Adhesion and spreading of mammalian cell

The rat bone marrow stem cells (BMSCs) were seeded on the samples(10 mm square plates) at a density of 5.0E4 cells/ml (1 ml/well). An hour later, the cells were washed with the phosphate buffer saline (PBS), fixed with 4% paraformaldehyde solution (Sigma) for 20 min at room temperature, and permeabilized with 0.1% (v/v) Triton X-100 (Amresco) for 2 min. Then, the cells were stained with FITC-Phalloidin (Sigma) and DAPI (Sigma) at room temperature in the dark. The F-actin and nuclei of the cells were examined on a fluorescence microscopy (Olympus GX71).

### Real-time polymerase chain reaction (RT-PCR)

The samples (three 20 mm square plates for each group) were placed in culture dishes (6 cm diameter, Nunc, Denmark) and BMSCs at densities of 1.0 E5 cell/well (culturing for 7 days) or 0.5 E5 cell/well (culturing for 14 days) were seeded on the samples. After culturing, the samples were rinsed three times with PBS, and the total RNA of the cells was extracted by a TRIZOL reagent (Invitrogen, USA). One milligram of the total RNA from each group was reverse transcribed into complementary DNA (cDNA) by Transcriptor First Strand cDNA Synthesis Kit according to the manufacturer’s protocols. Then, the expression of osteocalcin (OCN), osteopontin (OPN), alkaline phosphates (ALP), and bone morphogenetic protein 2 (BMP2) at mRNA levels were relatively quantified on the Real-Time PCR System (LightCycler 480, Roche, USA) by using the SYBR Green I PCR Kit and β-actin as the housekeeping gene for normalization. The 40 PCR cycles (95 °C for 10 sec. and 60 °C for 20 sec.) were started by denaturation at 95 °C for 30 sec. The primers for RT-PCR are listed in Table 2.

### Infection model of the rabbit tibia

All experiments were performed in accordance with relevant guidelines and regulations approved by the Animal Care and Experiment Committee of Sixth Peoples Hospital affiliated to Shanghai Jiao Tong University. Six female new zealand white rabbits (2.1–3.4 kg in weight) were used. Surgery was performed under general anesthesia by injecting of 3% pentobarbital (1 ml/1 Kg body wt) into the ear vein. The left tibia was exposed. Two holes with ~1 cm spacing and perpendicular to the tibia were drilled using a steel Kirschner wire (2.0 mm in diameter). The skin was sutured after placing the samples (cylindrical rods, 7 mm in length and 2 mm in diameter) into the holes. Then a steel Kirschner wire was inserted into the medullary cavity and pushed forward distally for smooth dilatation of the cavity. After removal, 1 ml of either saline or saline containing ATCC 43300 in a concentration of 1 × 10^4^ cfu/ml was injected by a microsyringe. After surgery, the animals were housed in separate cages and allowed to eat and drink ad libitum.

### Micro-computed tomography scanning

The animals were euthanized at the 6^th^ week post-surgery. Operated tibias (the whole) were dissected, harvested, and fixed in 10% buffered formalin. After the fixation, samples were scanned using high-resolution micro-computed tomography (microCT; Skyscan 1172, Skyscan, Belgium) at an image resolution of 18 μm. Reconstructed images in 2D and 3D were acquired using the software provided by the manufacturer.

### Histological evaluation

After 3D microCT scanning, the specimen was decalcified using 10% EDTA solution (pH 7.4) for 21 days, washed with running tap water for 3-4 h, transferred to a 75% ethanol solution, and embedded in paraffin. 10 μm sagittal sections of each specimen were collected. Hematoxylin and eosin (H&E) and Giemsa staining were used to assess the histology and the presence of bacteria around the implants^43^. The observation was conducted on a fluorescence microscope (Olympus GX71).

## Additional Information

**How to cite this article**: Cao, H. *et al.* Nano-thick calcium oxide armed titanium: boosts bone cells against methicillin-resistant staphylococcus aureus. *Sci. Rep.* **6**, 21761; doi: 10.1038/srep21761 (2016).

## Supplementary Material

Supporting Information

## Figures and Tables

**Table 1:** Parameters used in plasma ion immersion implantation (PIII).

	Target	Cathodic arc
Voltage pulse duration (μs)	500	500
Pulsing frequency (Hz)	7	7
Ion implantation voltage (kV)	−30	/
Ion implantation time (h)	1.5	/
Pressure (Pa)	5 × 10^−3^	/

**Table 2:** Primers for real-time polymerase chain reaction (RT-PCR).

Gene	Prime sequence (F, forward; R, reverse)	Product Size(bp)
OCN	F: GCCCTGACTGCATTCTGCCTCT	103
	R: TCACCACCTTACTGCCCTCCTG	
OPN	F: CCAAGCGTGGAAACACACAGCC	165
	R: GGCTTTGGAACTCGCCTGACTG	
ALP	F: CGTCTCCATGGTGGATTATGCT	209
	R: CCCAGGCACAGTGGTCAAG	
BMP-2	F: TGGGTTTGTGGTGGAAGTGGC	154
	R: TGGATGTCCTTTACCGTCGTG	
β-actin	F: CACCCGCGAGTACAACCTTC	207
	R: CCCATACCCACCATCACACC	
