# Supplementary material for: Nano-thick calcium oxide armed titanium: boosts bone cells against methicillin-resistant Staphylococcus aureus
Source: Sci Rep. 2016 Feb 22;6:21761. doi: 10.1038/srep21761 (PMC4761977; doi:10.1038/srep21761)
Supplement: Supporting Information [file srep21761-s1.doc]

Supporting Information

Nano-thick calcium oxide armed titanium: boosts bone cells against methicillin-resistant staphylococcus aureus

Huiliang Cao ^1^,*, Hui Qin ^2^,*, Yaochao Zhao ^2^,*, Guodong Jin ^1^, Tao Lu ^1^, Fanhao Meng ^1^, Xianlong Zhang ^2^, Xuanyong Liu ^1^

^1^ State Key Laboratory of High Performance Ceramics and Superfine Microstructure, Shanghai Institute of Ceramics, Chinese Academy of Sciences, Shanghai 200050, China.

^2^ Department of Orthopedics, Shanghai Sixth People’s Hospital, Shanghai Jiao Tong University, Shanghai 200233, China.

*These authors contributed equally to this work.

Correspondence and requests for materials should be addressed to

X. Z. ([zhangxianl197826@163.com](mailto:zhangxianl197826@163.com))

X. L. ([xyliu@mail.sic.ac.cn](mailto:xyliu@mail.sic.ac.cn) )


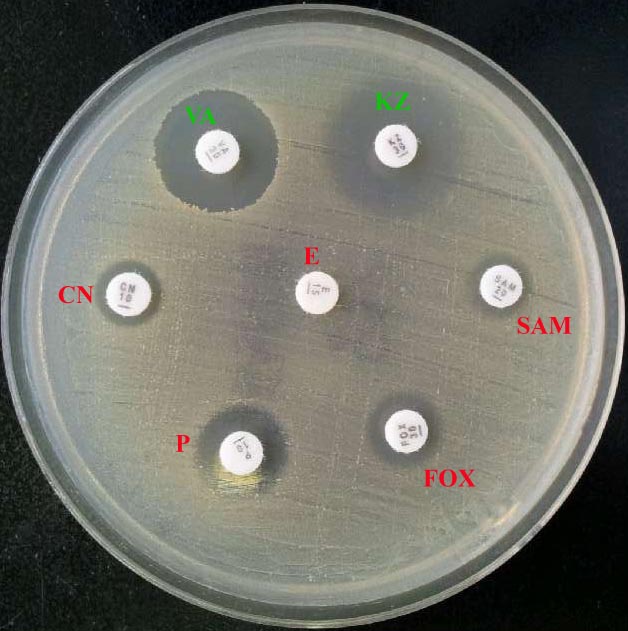


**Figure S1.** Susceptibility test for the bacterial strain (ATCC 43300), showing that the specie is resistant to gentamicin(CN), erythromycin(E), ampicillin-sulbactam(SAM), penicillin(P), and cefoxitin(FOX), but sensitive to vancomycin(VA) and cefazolin(KZ). The test was carried out accorrding to the BSAC standardized disc susceptibility testing method [*Andrews, J.M. BSAC standardized disc susceptibility testing method (version 8). J. Antimicrob. Chemother.* ***64****, 454-489(2009)*].


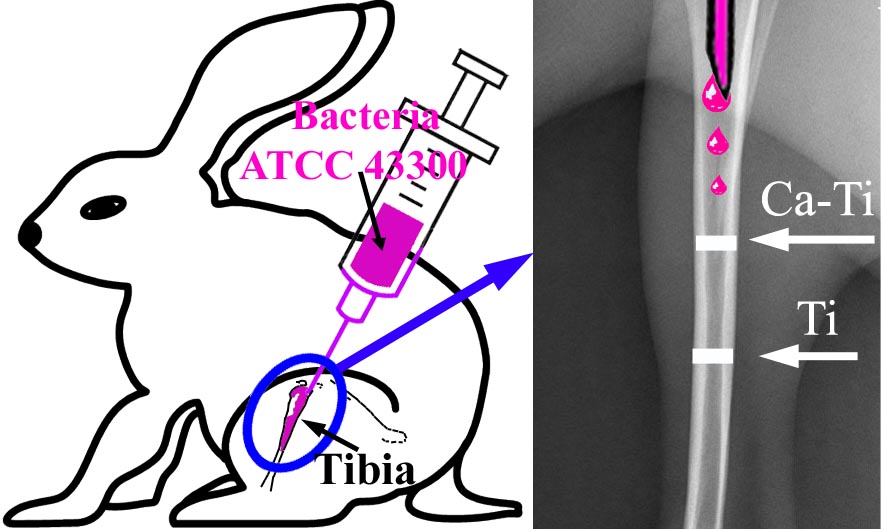


**Figure S2.**  Schematic of surgical procedures and the relative position of the sample groups. n=6, one of them was not contaminated with bacteria, the other five were injected of MRSA. The cartoon was drawn by H.C.
